# Mapping Evidence on Care Interventions for People Living With Motor Neurone Disease: A Protocol for a Living Evidence and Gap Map

**DOI:** 10.1002/gin2.70062

**Published:** 2026-02-04

**Authors:** Danielle Pollock, Cindy Stern, Melissa Bond, Timothy Hugh Barker, Sabira Hasanoff, Grace Holland, Ines Semendric, Anna Fragkoudi, Nipun Shrestha, Sebastian Cole Facchin‐Young, Abigail Molly Day‐Sharman, Lemma Negesa Bulto, Lara Stollery, Sitasma Sharma, Camille Schubert, Lynne Giles, Jay Beasley‐Hall, Tracy Merlin, Julie Labra, Steve Vucic, Taryn Hunt, Zachary Munn

**Affiliations:** ^1^ Health Evidence Synthesis, Recommendations and Impact (HESRI), School of Public Health, College of Health Adelaide University Adelaide Australia; ^2^ EPPI Centre, University College London UK; ^3^ Knowledge Center for Education University of Stavanger Stavanger Norway; ^4^ School of Biomedicine, Faculty of Health and Medical Sciences The University of Adelaide Adelaide Australia; ^5^ Adelaide Health Technology Assessment (AHTA), School of Public Health, Faculty of Health and Medical Sciences The University of Adelaide Adelaide Australia; ^6^ School of Public Health, Faculty of Health and Medical Sciences The University of Adelaide Adelaide Australia; ^7^ University Library The University of Adelaide Adelaide Australia; ^8^ MND Australia, Australian Capital Territory Canberra Australia; ^9^ Brain and Nerve Research Centre, Concord Clinical School The University of Sydney Sydney Australia; ^10^ Person With Lived Experience of MND

**Keywords:** clinical management, evidence and gap map, health‐related quality of life, motor neurone disease, review

## Abstract

**Objective:**

To map the available evidence informing the care and management of people living with motor neurone disease (MND), including evidence pertaining to carers, asymptomatic genetic carriers, family, friends and healthcare professionals caring for people with MND.

**Introduction:**

MND is a devasting neurodegenerative disease that has no cure and eventually leads to paralysis, progressive speech difficulties and respiratory failure. There are a plethora of interventions to support people living with MND, however, information regarding these interventions is often not organised in a manner that best supports the MND community. An evidence and gap map will provide a visual presentation that highlights the existing evidence that is available to support specific interventions, and indicate where there is no evidence. This will help inform future guideline and research efforts.

**Inclusion Criteria:**

Studies if they describe care interventions for people living with MND, carers, asymptomatic genetic carriers, family, friends and healthcare professionals caring for people with MND. Peer‐reviewed randomised controlled trials, and nonrandomised studies of interventions, systematic reviews and qualitative studies describing the experience of an intervention will be included.

**Methods:**

An evidence and gap map will be conducted according to Campbell guidance and JBI guidance for scoping reviews. A comprehensive search of academic databases and clinical trial registries will be performed to identify eligible studies. An evidence inclusion framework has been developed with members of the MND community. Data screening and extraction will be independently performed by two reviewers. The evidence and gap map will be presented using EPPI Reviewer.

**Review Registration:**

This protocol has been uploaded to the Australian Motor Neurone Disease (MND) Guideline Open Science Framework page (https://osf.io/c2ezm/).

## Summary

- Motor neurone disease (MND) is a devasting neurodegenerative disease that has no cure.
- There is currently no Australian care guideline for MND.
- This evidence and gap map will be conducted according to Campbell guidance and JBI guidance for scoping reviews.
- Consultation with interest‐holders, including those living with MND will occur.
- This living evidence and gap map will act as a centralised surveillance of evidence for the Australian MND Care guidelines.

## 1 Background

Motor Neurone Disease (MND) is a devasting neurodegenerative disease that has no cure and eventually leads to paralysis, progressive speech difficulties and respiratory failure [1]. MND presents great challenges to the families and carers of people living with MND, as the disease progressively diminishes the person's ability to complete daily tasks, such as showering, writing, communicating and walking [2]. In 2019, the global incidence of people living with MND was 268,674 individuals, and since 1990, this incidence has risen 1.9%. This increase since 1990 is particularly in high‐income regions (North America, Western Europe and Australasia), to which over half of these cases are attributed [3]. In Australia, MND affects approximately 2600 people at any given time, with two Australians dying due to MND every day [4, 5, 6]. Despite the prevalence of MND, the only available treatment in Australia to slow progression is riluzole, which has been shown to extend survival by 6–19 months [2].

Previous research into MND has primarily focused on the identification of risk factors and finding a cure [2]. However, until a cure is found, there is a need to support and provide the best available care for people living with MND [2]. Caring for someone living with MND is complex and requires an interdisciplinary approach involving a range of healthcare professionals, support workers and family support [7, 8]. Interventions to support the provision of MND care exist, but there are limited best‐practice guidelines. No formal guidelines for MND care in Australia currently exist. High‐quality guidelines can facilitate early diagnosis, and inform multidisciplinary care, symptom management and supportive interventions tailored to the individual needs of MND patients [9]. Additionally, they can aid in the provision of timely access to assistive technologies and palliative care services, which are essential components of comprehensive MND management [10]. By providing recommended care approaches based on the evidence, balanced with individual preferences and values, high‐quality guidelines can enhance quality of life, prolong survival and alleviate suffering for individuals living with MND. They can also inform government bodies and providers, such as the National Disability Insurance Scheme, as to what supports and assistive technologies are indicated for people living with MND.

FightMND (a not‐for‐profit organisation) recognised the need for standardised care and subsequently funded the development of the Australian MND Guideline to improve the quality of life for Australians living with MND, their families and their carers. The development of good‐quality clinical practice guidelines is a complex, time‐consuming process requiring substantial financial and intellectual investment [11, 12, 13, 14, 15]. Guideline recommendations must be based on transparent and rigorous evidence synthesis [15]. To support this endeavour, the developers of the Australian MND Guideline will develop a living ‘evidence and gap map’ (EGM), to support centralised surveillance of evidence in MND care.

The EGM will be hosted on an interactive online platform, updated monthly by the authorship team. The EGM will provide a visual presentation of available evidence informing the care of those living with MND, thus allowing clinicians, researchers, decision makers and people impacted by MND to efficiently identify where the existing evidence is available and where there is no evidence [16, 17]. This EGM will form the evidence foundation for the Australian MND guideline, as well as future research efforts within the MND community.

## 2 Methods

This EGM will be conducted according to Campbell guidance [16] and JBI guidance for scoping reviews [18]. Currently, there is no reporting standard available for EGMs, however, some are under development, such as the Preferred Reporting Items for Evidence Mapping (PRITEM) [19], and the preferred reporting Items for systematic reviews and meta‐analysis extension for evidence and gap maps (PRISMA‐EGMs) [20]. As this EGM will be living, the reporting will be guided by the preferred reporting items for systematic reviews and meta‐analysis extension for living systematic reviews (PRISMA‐LSR) statement [21]. This protocol has been uploaded to the Australian motor neurone disease (MND) Guideline Open Science Framework page (https://osf.io/c2ezm/).

## 3 Consultation With Knowledge Users

This review has been commissioned by FightMND as part of a broader project to develop the Australian MND guideline. The inclusion of those with lived experience of MND (including people living with MND, carers and genetic carriers), researchers, decision makers and healthcare professionals will ensure a comprehensive and useful EGM that can be applied within the community.

This EGM will be developed in collaboration with the MND community, including:

- Co‐develop the EGM evidence inclusion framework.
- Assist with the identification of relevant evidence sources.
- Assist in the development of the data extraction table to be used in the conduct of the screening, extraction and analysis of results.
- Provide an interpretation of the findings of this EGM.

### 3.1 Review Question

What is the available evidence informing the care of those living with MND?

### 3.2 Eligibility Criteria

Although EGMs typically follow a population, intervention, comparator and outcome (PICO) framework, the comparator can be excluded if it is not required [16]. As there are numerous comparators to be considered within these interventions and they are not the focus of this EGM, comparators will not be used to guide the evidence collection.

This EGM will consider studies focusing on the following populations:

1. People who are living with MND
  Adults (18+) diagnosed with any type of MND, such as amyotrophic lateral sclerosis (ALS), progressive bulbar palsy (PBP), progressive muscular atrophy (PMA), primary lateral sclerosis (PLS), MND with frontal temporal dementia (MND/FTD), Kennedy's disease/spinal and bulbar muscular atrophy (SBMA).
2. Asymptomatic carriers of MND‐associated genes
  This EGM will include people who are genetic carriers of known MND‐related genes. To be eligible for inclusion this population must have undertaken genetic testing and be found to be a carrier of a recognised relevant genetic variant. Potential genes include: *C9orf72*, *SOD1*, *FUS* or *TARDBP* [22].
3. Carers supporting, or who have previously supported, those living with MND
  Carers can include family or nonfamily support (paid or unpaid). This can include past or present carers.
4. Spouses, family members and friends of people who are living with MND
  Immediate family members (i.e., children, siblings, parents), extended family (e.g., grandparents, nieces, nephews, cousins) or friends are eligible for inclusion.
5. Healthcare professionals working with people who are living with MND

Any professional who provides health care; this includes medical, physical, psychological or social support of people who are living with MND. Such professionals might include medical specialists, general practitioners, nurses or allied health practitioners. Disability, aged and support workers will also be included.

Studies which investigate other progressive neurological disorders alongside MND will be assessed for eligibility. However, only studies with data relevant to MND, that can be extracted, will be eligible for inclusion.

#### 3.2.1 Intervention

This EGM will provide a holistic representation of the available interventions which aim to provide care to improve the health and well‐being of a person living with MND, whether diagnosed or an asymptomatic carrier. It will consider any interventions spanning the MND diagnostic pathway, as well as the care‐management pathway (following a confirmed diagnosis of MND or confirmation that a person is a genetic carrier).

Interventions may include genetic screening, care approaches, symptom management, palliative care or advance care planning. Approaches may be medical, surgical, pharmacological, physical, social, emotional, psychological, spiritual or educational, amongst others and may be utilised as a single or combined, intervention. Interventions may be provided by people living with MND (self‐care), carers, spouses, family members, friends or healthcare professionals. Interventions which are multicomponent that is, includes clinical and psychological will be coded for each component.

#### 3.2.2 Context

This EGM will incorporate evidence from all countries, health systems and care settings (e.g., community, primary, secondary and tertiary care).

#### 3.2.3 Outcomes

Through preliminary investigation of the evidence, we have identified numerous outcomes that will be initially included within the EGM framework. These include outcomes related to:

– adherence to the intervention,
– education and awareness
– biological indicators
– cognitive performance,
– economic impact
– physical health and function
– psychosocial wellbeing
– quality of life and
– survival

Members within the MND community will be asked to review the current framework (Supporting File S1) and suggest any other relevant outcomes to be added.

We will not be extracting *outcome measure tools*, for example, we will extract ‘difficulty with swallowing’ (e.g., dysphagia) as an outcome that is assessed, but would not list the tools used to measure dysphagia (such as the Dysphagia Outcomes and Severity Rating Scale) [23].

#### 3.2.4 Study Design

This EGM will consider both experimental and quasi‐experimental study designs including randomised controlled trials (and variants), various nonrandomised studies including before and after studies and interrupted time‐series studies. In addition, observational studies including prospective and retrospective cohort studies, case‐control studies and cross‐sectional studies will be considered for inclusion. This review will also consider descriptive observational study designs including case series, and descriptive cross‐sectional studies for inclusion if related to an intervention. Case reports will not be included within this EGM.

This EGM will also include mixed methods primary studies, and qualitative studies of any study design, which explore the stated population's experiences related to specific treatment options/modalities/interventions. Qualitative studies exploring the ‘general’ experiences of living with, or caring for someone, with MND, will not be included.

Systematic reviews and overviews (labelled as such by the author) which explore the effectiveness or experience with an intervention will be included. These reviews may be quantitative, qualitative or mixed methods.

Registered trials will be assessed for eligibility to be included in this EGM in the future and flagged as a study in progress.

### 3.3 Search Strategy

A two‐step search strategy will initially be utilised in this review, and once the review enters ‘living’ mode all additional searches will occur through OpenAlex. Initially, a medical librarian (J.B.H.) developed a limited search of PubMed via NCBI. The text words contained in the titles and abstracts of relevant articles, and the index terms used to describe the articles were used to develop a full search strategy for MEDLINE (Ovid). The full search strategy, including all identified keywords and index terms, was then adapted for each included database and/or information source (see Supporting File S1). Due to the expected high volume of eligible studies, a backward and forward citation searching of the research included will not be feasible.

The databases to be searched include the Cochrane Central Register of Controlled Trials (CENTRAL); MEDLINE (Ovid); EMBASE (Ovid); and PsycINFO (Ovid). Further searching to identify clinical trials will be performed through ClinicalTrials. gov (www.ClinicalTrials.gov/) and The WHO's International Clinical Trials Registry Platform (WHO ICTRP) (www.who.int.ictrp).

There will be no date limitations. Studies published in any language will be assessed for eligibility. Abstracts presented in languages other than English will be translated using Google Translate or DeepL to be assessed for eligibility. If these studies are considered eligible at this stage, then the full‐text will also be imported into Google Translate or DeepL. If these programs are unable to translate these studies into English, all attempts will be made to identify a person who will be able to translate the full‐text. If studies cannot be translated, they will be excluded.

All search strategies will be submitted as Supporting materials with the published EGM.

### 3.4 Study Selection

After completion of the search, all identified citations will be collated and uploaded into EndNote v.X20 (Clarivate Analytics, PA, USA). Duplicates will be removed within Endnote. Deduplicated evidence sources will then be imported into the evidence synthesis software Evidence for Policy and Practice Information (EPPI) reviewer to commence screening [24].

Screening of title and abstracts will be performed and then studies that may be eligible will have their full‐text retrieved to facilitate a second round of screening at full‐text level. Piloting of the screening process will occur before title and abstract screening commences and prior to reviewing full texts for eligibility. Piloting will involve each reviewer independently screening 50 titles and abstracts as well as 10 full‐text evidence sources. Each evidence source during title and abstract and full‐text screening will be reviewed against the eligibility criteria by two reviewers independently. More piloting, discussions and clarification to the screening guidance will occur if > 70% agreement between reviewers has not been achieved. To help facilitate the title and abstract screening process, priority screening within EPPI Reviewer will be activated after the initial piloting phase and after 10 items have been agreed to be included [25]. This screening approach automatically prioritises relevant title and abstracts based on previous decisions.

Weekly meetings as a team will occur to discuss decision/screening conflicts. Any disagreements that arise between the reviewers at each stage of the selection process will be resolved through discussion within these meetings or with a third reviewer. As prescribed, the search results and study inclusions will be reported in full in the final review and presented in a PRISMA flow diagram [26]. Reasons for exclusion of full‐text documents that do not meet the inclusion criteria will be recorded and reported in the final review.

### 3.5 Assessment of Risk of Bias and Methodological Quality

EGMs can perform risk of bias assessments or critical appraisal on the included studies [27]. However, the intention of this EGM is not to provide an assessment of the bias or quality of the included studies, but to determine where evidence exists. Therefore, this EGM will not be including a risk of bias assessment or assessment of the methodological quality of the included studies.

### 3.6 EGM Inclusion Framework

The development of the EGM inclusion framework is a crucial part of the process. Within this EGM, all attempts have been made to develop a comprehensive and deductive framework prior to the extraction of data from the studies. The EGM inclusion framework has been developed through preliminary investigation of the evidence and community consultation. Table 1 provides a simple example of some of the elements of the EGM framework, with the full framework available in the Supporting materials. However, there is scope for further outcomes to be included if they are identified within studies. If a new outcome is identified, the reviewer will stop extracting that study, notify the lead reviewer (D.P.), who will then assess if it should be added to the framework where the reviewer can then continue extracting.

**Table 1:** Example of EGM framework.

Population	Intervention	Outcome	Types of studies
People who are living with MND	Invasive ventilatory support tracheostomy, mechanical ventilation, translaryngeal intubation, Laryngectomy, NeuRx® Diaphragm Pacing System™ (DPS), Laryngeal closure	Quality of life and symptoms Communication Sleep disorders Nutrition Pain Psychosocial impact Saliva control (sialorrhea) Symptom management Fatigue	RCTS Nonrandomised studies of interventions Systematic reviews and overviews Qualitative studies Trial in Progress
• MND • ALS • PBR • PMA • PLS • MND/FTD/SBMA			
Genetic carriers of known MND genes Carers supporting those living with MND Family members and friends of people who are living with MND Health care professionals working with people who are living with MND Priority Populations First nations poples Culturally and Linguistically diverse peoples Rural/regional specific Australian			

### 3.7 Data Extraction

Extraction will occur in EPPI Reviewer using the EGM inclusion framework, which has been specifically designed for this review (see Supporting File S1). Prior to starting data extraction, reviewers will be asked to pilot data from 10 studies. During piloting, the extraction form may be calibrated based on the feedback received by the reviewers to ensure that all necessary information has been collected. Once piloting has been finalised, each study will be extracted independently by two reviewers. If there are any modifications to the framework, it will be declared in the published scoping review. Any discrepancies between individual extraction of the same evidence source will be resolved through discussion or with an additional reviewer.

### 3.8 EGM Development

Once all data have been extracted, the data will be exported from EPPI Reviewer as a JSON (JavaScript Object Notation) file and imported into EPPI Mapper [28]. Display and filter options will be chosen from the framework (see Table 1), and a HTML file will be downloaded and subsequently hosted online for easy stakeholder access. Depending on interest holders feedback, multiple EGMs might be created to explore the evidence from different angles.

An openly accessible web database of all included studies and associated coding decisions will also be created using EPPI visualiser, to provide interest holders with the opportunity to produce their own frequency and crosstabulation charts (downloadable as a.ris file of the included studies), or explore the coding in a more nuanced way.

The results of the EGM will be narratively described within a report.

### 3.9 Living EGM

The EGM will be updated monthly for the duration of the Australian MND Guideline, which has an expected completion of December 2026. Following the inclusion of all items at full‐text level from the initial search, the OpenAlex tools [29] within EPPI Reviewer will be used to identify any study reports that recommend, cite or are cited by items already included, which will then be imported into the review for screening. An automated OpenAlex search will be setup using the ‘Keep up to date’ feature, which will deliver a list of potential items monthly. Where databases allow, automated emails will also be set up to provide a weekly update to the project team on new research that matches the search parameters.

Following screening of full‐text for the baseline review, a stratified random assignment of records will be undertaken to allocate the records to one of three groups—a ‘training set’, a ‘calibration set’ and an ‘evaluation’ set, at a ratio of 3:1:1. A binary machine learning classifier will then be trained within EPPI Reviewer using the ‘training set’ records. A classification threshold score will be determined using the ‘calibration set’ records, to meet a recall > 0.95, and will be applied to the ‘evaluation set’ records. The custom classifier will then be rebuilt using 100% of all screened records, and then deployed in further continual updating of the living map to discard new records (those located by the regular email alerts or by an automated monthly OpenAlex search) that score below the established threshold before screening), thereby reducing screening workload, with minimal risk of ‘losing’ eligible studies [30]. A new EGM will be generated and uploaded to the project website once every month, updated with any new records found.

#### 3.9.1 Study Within a Review (SWAR)

A SWAR is a study undertaken while doing an evidence synthesis, to evaluate the effectiveness of a new methodological approach to a particular process [31]. During this project, a retrospective SWAR will be undertaken after full‐text screening has been completed using the initial search results to compare the efficiency and accuracy of screening using the GPT‐4.1 large language model (LLM) integration within EPPI reviewer as opposed to human screening. This SWAR will use the method outlined in the published protocol [31] and form part of a living SWAR to assess the performance of generative AI tools for screening. Although the number of studies evaluating generative AI for screening is increasing [32], their methodological reporting standards are not high enough in many cases to allow replication. Conducting several SWARs using the same method with diverse datasets will enable the authors to conduct a cumulative meta‐analysis of the SWAR replications, thereby building a more robust evidence base for the use of generative AI tools for eligibility screening.

As outlined in the protocol, expected primary outcomes will be recall, precision, conflict resolutions and full‐text screening, with secondary outcomes including comparisons of raw % agreement, Cohen's Kappa (pairwise comparisons between all interventions), comparisons of true and false positives and comparisons of true and false negatives. The SWAR will not interfere with the EGM development.

### 3.10 Ethics and Dissemination

As this EGM is a review of secondary evidence it does not require ethics approval. No individual‐level participant data will be collected and all information used will be in the public domain. All study files (search strategies, data extraction forms and the EGM, etc.) will be made available via a publicly available project space using open science platforms.

## Author Contributions

**Danielle Pollock:** conceptualisation, methodology, project administration, resources, funding acquisition, writing – review and editing; writing – original draft; funding acquisition. **Cindy Stern:** conceptualisation, methodology, project administration, resources, writing – original draft, writing – review and editing. **Melissa Bond:** conceptualization, methodology, project administration, resources, writing – original draft, writing – review and editing. **Timothy Hugh Barker:** conceptualization, methodology, funding acquisition, writing – original draft, writing – review and editing. **Sabira Hasanoff:** conceptualisation, writing – original draft, writing – review and editing. **Grace Holland:** Conceptualization, writing – original draft, writing – review and editing. **Ines Semendric:** conceptualisation, writing – original draft, writing – review and editing. **Nipun Shrestha:** conceptualisation, writing – original draft, writing – review and editing. **Anna Fragkoudi:** conceptualisation, writing – original draft, writing – review and editing. **Sebastian Cole Facchin‐Young:** conceptualisation, writing – original draft, writing – review and editing. **Abigail Molly Day‐Sharman:** conceptualisation, writing – original draft, writing – review and editing. **Lemma Negesa Bulto:** conceptualisation, writing – original draft, writing – review and editing. **Lara Stollery:** conceptualisation, writing – original draft, writing – review and editing. **Sitasma Sharma:** conceptualisation, writing – original draft, writing – review and editing. **Camille Schubert:** conceptualisation, writing – original draft, writing – review and editing. **Lynne Giles:** conceptualisation, writing – original draft, writing – review and editing. **Jay Beasley‐Hall:** conceptualisation, methodology, writing – original draft, writing – review and editing. **Tracy Merlin:** conceptualisation, funding acquisition, writing – original draft, writing – review and editing. **Julie Labra:** conceptualisation, writing – original draft, writing – review and editing. **Steve Vucic:** conceptualisation, writing – original draft, writing – review and editing. **Taryn Hunt:** conceptualisation, writing – original draft, writing – review and editing. **Zachary Munn:** conceptualisation, methodology, project administration, resources, writing – original draft, writing – review and editing, funding acquisition. All authors read and approved the final manuscript.

## Conflicts of Interest

Cindy Stern, Danielle Pollock, Grace Holland, Nipun Shrestha and Ines Semendric are members of the Adelaide GRADE Centre. Sabira Hasanoff is the coordinator of the Adelaide GRADE Centre. Timothy Barker is Deputy Director of the Adelaide GRADE Centre. Zachary Munn is Director of the Adelaide GRADE Centre and board member of the Guideline International Network. Danielle Pollock and Zachary Munn are members of the Campbell NAVIGATE group. Danielle Pollock is the chair of the Guideline International Network Australian and New Zealand Regional group.

## Supporting information

Supplementary_for_submission.

## Data Availability

All data for this protocol, and for the entire Australian MND Guideline, are available on the Open Science Framework (OSF) at https://osf.io/c2ezm/.

## References

[1] C. J. McDermott and P. J. Shaw , “Diagnosis and Management of Motor Neurone Disease,” BMJ 336, no. 7645 (2008): 658–662. 18356234 10.1136/bmj.39493.511759.BE PMC2270983

[2] C. A. Wood‐Allum , “Unanswered Questions and Barriers to Research in the Palliative Care of Motor Neurone Disease Patients,” Journal of Palliative Care 30, no. 4 (2014): 302–306. 25962266

[3] J. Park , J.‐E. Kim , and T.‐J. Song , “The Global Burden of Motor Neuron Disease: An Analysis of the 2019 Global Burden of Disease Study,” Frontiers in Neurology 13 (2022): 1–15, 10.3389/fneur.2022.864339. PMC9068990 35528743

[4] R. Sheean and G. Thomas , The MND Collective (MND Australia, 2023).

[5] J. Luker , R. Woodman , and D. Schultz , “The Incidence and Prevalence of Motor Neurone Disease in South Australia,” Amyotrophic Lateral Sclerosis and Frontotemporal Degeneration 24, no. 3–4 (2023): 195–202. 35934980 10.1080/21678421.2022.2108326

[6] T. Dharmadasa , R. D. Henderson , P. S. Talman , et al., “Motor Neurone Disease: Progress and Challenges,” Medical Journal of Australia 206, no. 8 (2017): 357–362. 28446118 10.5694/mja16.01063

[7] N. G. Simon , W. Huynh , S. Vucic , K. Talbot , and M. C. Kiernan , “Motor Neuron Disease: Current Management and Future Prospects,” Internal Medicine Journal 45, no. 10 (2015): 1005–1013. 26429216 10.1111/imj.12874

[8] U. E. Williams , E. E. Philip‐Ephraim , and S. K. Oparah , “Multidisciplinary Interventions in Motor Neuron Disease,” Journal of Neurodegenerative Diseases 2014, no. 1 (2014): 435164. 26317009 10.1155/2014/435164 PMC4437278

[9] M. Lugtenberg , J. S. Burgers , and G. P. Westert , “Effects of Evidence‐Based Clinical Practice Guidelines on Quality of Care: A Systematic Review,” Quality and Safety in Health Care 18, no. 5 (2009): 385–392. 19812102 10.1136/qshc.2008.028043

[10] N. Layton , A. Spann , M. Khan , et al., “Guidelines for Assistive Technology Service Provision—A Scoping Review,” Disability and Rehabilitation. Assistive Technology 19, no. 8 (2024): 2806–2817. 38476029 10.1080/17483107.2024.2327515

[11] National Health and Medical Research Council , Australian Clinical Practice Guidelines 2014 Annual Report Canberra, 2014, https://www.nhmrc.gov.au/sites/default/files/documents/reports/clinical-practice-guidelines-annual-report-2014.pdf.

[12] National Health and Medical Research Council , Procedures and Requirements for Mmeeting the 2011 NHMRC Standard for Clinical Practice Guidelines 2011, https://www.nhmrc.gov.au/sites/default/files/documents/reports/clinical%20guidelines/Procedures-and-requirements-for-meeting-the-2011-NHMRC-standard-for-clinical-practice-guidelines%2801%29.pdf.

[13] National Health and Medical Research Council , Reporting on Australian Clinical Practice Guidelines 2016, https://www.nhmrc.gov.au/guidelines-publications/information-guideline-developers/reporting-australian-clinical-practice.

[14] Z. Munn , S. Twaddle , D. Service , et al., “Developing Guidelines Before, During, and After the COVID‐19 Pandemic,” Annals of Internal Medicine 173, no. 12 (2020): 1012–1014. 32931327 10.7326/M20-4907 PMC7505021

[15] H. J. Schünemann , W. Wiercioch , I. Etxeandia , et al., “Guidelines 2.0: Systematic Development of a Comprehensive Checklist for a Successful Guideline Enterprise,” Canadian Medical Association Journal 186, no. 3 (2014): E123–E142. 24344144 10.1503/cmaj.131237 PMC3928232

[16] H. White , B. Albers , M. Gaarder , et al., “Guidance for Producing a Campbell Evidence and Gap Map,” Campbell Systematic Reviews 16, no. 4 (2020): e1125. 37016607 10.1002/cl2.1125 PMC8356343

[17] A. Saran and H. White , “Evidence and Gap Maps: A Comparison of Different Approaches,” Campbell Systematic Reviews 14, no. 1 (2018): 1–38. 10.4073/cmdp.2018.2 PMC8428058 37131398

[18] M. D. J. Peters , C. Marnie , A. C. Tricco , et al., “Updated Methodological Guidance for the Conduct of Scoping Reviews,” JBI Evidence Synthesis 18, no. 10 (2020): 2119–2126. 33038124 10.11124/JBIES-20-00167

[19] Y. Li , E. Ghogomu , M. Gaarder , et al., “The Development of PRITEM Reporting Guideline for Mapping Reviews,” OSF (2024), https://osf.io/6ysk8/overview.

[20] F. Campbell , PRISMA‐EGMs–PRISMA‐Evidence and Gap Maps. EQUATOR NETWORK, 2023, https://www.equator-network.org/library/reporting-guidelines-under-development/reporting-guidelines-under-development-for-systematic-reviews/#EGM.

[21] E. A. Akl , J. Khabsa , C. Iannizzi , et al., “Extension of the PRISMA 2020 Statement for Living Systematic Reviews (PRISMA‐LSR): Checklist and Explanation,” BMJ 387 (2024): e079183. 39562017 10.1136/bmj-2024-079183 PMC12036629

[22] H. A. Black , D. J. Leighton , E. M. Cleary , et al., “Genetic Epidemiology of Motor Neuron Disease‐Associated Variants in the Scottish Population,” Neurobiology of Aging 51 (2017): 178.e11‐e20. 10.1016/j.neurobiolaging.2016.12.013 PMC5302213 28089114

[23] K. H. O'Neil , M. Purdy , J. Falk , and L. Gallo , “The Dysphagia Outcome and Severity Scale,” Dysphagia 14, no. 3 (1999): 139–145. 10341109 10.1007/PL00009595

[24] J. Thomas , S. Graziosi , J. Brunton , et al., EPPI‐Reviewer: Advanced Software for Systematic Reviews, Maps and Evidence Synthesis (EPPI Centre, UCL Social Research Institute, University College London, 2023).

[25] S. Waffenschmidt , W. Sieben , T. Jakubeit , et al., “Increasing the Efficiency of Study Selection for Systematic Reviews Using Prioritization Tools and a Single‐Screening Approach,” Systematic Reviews 12, no. 1 (2023): 161. 37705060 10.1186/s13643-023-02334-x PMC10500815

[26] M. J. Page , J. E. McKenzie , P. M. Bossuyt , et al., “The PRISMA 2020 Statement: An Updated Guideline for Reporting Systematic Reviews,” BMJ 372 (2021): n71. 33782057 10.1136/bmj.n71 PMC8005924

[27] F. Campbell , A. C. Tricco , Z. Munn , et al., “Mapping Reviews, Scoping Reviews, and Evidence and Gap Maps (EGMS): The Same but Different— the ‘Big Picture’ Review Family,” Systematic Reviews 12, no. 1 (2023): 45. 36918977 10.1186/s13643-023-02178-5 PMC10014395

[28] Centre DSFaE ., EPPI‐Mapper (EPPI Centre, UCL Social Research Institute, University College London, 2023).

[29] J. Priem , H. Piwowar , and R. Orr , “OpenAlex: A Fully‐Open Index of Scholarly Works, Authors, Venues, Institutions, and Concepts,” 2022, arXiv preprint arXiv:220501833.

[30] C. Stansfield , G. Stokes , and J. Thomas , “Applying Machine Classifiers to Update Searches: Analysis From Two Case Studies,” Research Synthesis Methods 13, no. 1 (2022): 121–133. 34747151 10.1002/jrsm.1537 PMC9299040

[31] D. Devane , N. N. Burke , S. Treweek , et al., “Study Within a Review (SWAR),” Journal of Evidence‐Based Medicine 15, no. 4 (2022): 328–332. 36513956 10.1111/jebm.12505 PMC10107874

[32] P. Scheelbeek , M. Bond , M. Callaghan , et al., Digital Evidence Synthesis Tools for Climate & Health (Trust TW, 2024).

