# Supplementary material for: Mapping Evidence on Care Interventions for People Living With Motor Neurone Disease: A Protocol for a Living Evidence and Gap Map
Source: Clin Public Health Guidel. 2026 Feb 4;3(2):e70062. doi: 10.1002/gin2.70062 (PMC13563322; doi:10.1002/gin2.70062)
Supplement: Supplementary file 1 [file GIN2-3-e70062-s001.docx]

# Supplementary Materials

# Search Strategy

**Medline (PubMed via Ovid) (25/02/2025)**

| 1  MND | Exp motor neuron disease OR Spastic Paraplegia, hereditary.sh OR (Gehrig* disease OR motor neuron* disease* OR progressive bulbar pals* OR progressive muscular atroph* OR spinal muscular atroph* OR hereditary spastic parapares* OR "spinal and bulbar muscular atroph*" OR Kennedy* disease* OR Kennedy* syndrome* OR bulbo-spinal atroph* or flail arm ALS OR flail leg ALS or flail arm syndrome OR anterior horn cell disease* OR charcot disease* OR guam disease* OR bulbar pals* OR fazio-londe* disease* OR fazio-londe* syndrome* OR spinal amyotroph* OR spinal muscular atroph* OR bulbospinal neuronopath* OR hereditary motor neuronopath* OR infantile muscular atroph* OR juvenile muscular atroph* OR infantile acute form SMA OR werdnig hoffman* disease* OR motoneuron disease* OR motor system disease* OR ALS Dementia OR chinese paralytic syndrome* OR Aran Duchenne OR Duchennen Aran OR hereditary spastic paraplegia* OR hereditary autosomal dominant spastic paraplegia* OR hereditary autosomal recessive spastic paraplegia* OR "hereditary motor and sensory neuropath*" OR hereditary x linked recessive spastic paraplegia* OR hypertrophic motor sensory neuropathy spastic paraplegia* OR spastic paraplegia type 2 OR Charcot Marie OR Charcot Hoffman OR hereditary motor sensory neuropath* OR Dejerine sottas OR familial spastic para* OR "hereditary motor and sensory neuropath*" OR hereditary sensorimotor neuropath* OR "hereditary sensory and motor neuropath*" OR neurogenic amyotroph* OR neurogenic atroph* OR neurogenic muscle atroph* OR neurogenic muscle syndrome* OR peroneal atroph* OR perone* muscle atroph* OR perone* muscular atroph* OR spastic hereditary paraglegia OR Struemell* disease* OR Struempell Lorrain OR Strumpel* disease* OR spinal amyotroph* OR bulbospinal neuronopath* OR distal spinal muscular atroph* OR hereditary motor neuronaopath* OR myelopathic muscular atroph* OR Spinobulbar muscular atroph* OR bulbo-spinal atroph* OR MND OR lateral scleros*).ti,ab,kw OR lateral scleros*.kf | 63420 |
| --- | --- | --- |
| 2  Care Interventions | Exp patient care management OR exp therapeutics OR exp social support OR exp surgical procedures, operative OR exp psychology OR exp psychiatry OR exp psychotherapy OR ((care OR treat* OR support* OR assist* OR plan* OR program* OR service* OR interven* OR therap* OR manag* OR approach* OR procedur* OR healthcare) adj10 (pharm* OR medic* OR drug* OR physical* OR social* OR emotion* OR psychosocial* OR spiritual* OR educat* OR palliative OR "end of life" OR hospice* OR tech* OR terminal OR psych* OR surg* OR non-surg* OR exercise* OR multidisciplin* OR multi-disciplin* OR mental*)).ti,ab,kw | 10848923 |
| 3  Study types | epidemiologic methods.sh OR epidemiologic studies.sh OR observational study.sh OR observational studies as topic.sh OR clinical studies as topic.sh OR controlled before-after studies.sh OR cross-sectional studies.sh OR historically controlled study.sh OR interrupted time series analysis.sh OR exp seroepidemiologic studies OR national longitudinal study of adolescent health.sh OR cohort studies.sh OR cohort analysis.sh OR longitudinal studies.sh OR longitudinal study.sh OR prospective studies.sh OR prospective study.sh OR follow-up studies.sh OR follow up.sh OR followup studies.sh OR retrospective studies.sh OR retrospective study.sh OR case-control studies.sh OR exp case control study OR cross-sectional study.sh OR observational study.sh OR quasi experimental methods.sh OR quasi experimental study.sh OR single-case studies as topic.sh OR (observational study or validation studies or clinical study).pt OR (observational adj3 (study or studies or design or analysis or analyses)).ti,ab,kf OR cohort*.ti,ab,kf OR (prospective adj7 (study or studies or design or analysis or analyses)).ti,ab,kf OR ((follow up or followup) adj7 (study or studies or design or analysis or analyses)).ti,ab,kf OR ((longitudinal or longterm or (long adj term)) adj7 (study or studies or design or analysis or analyses or data)).ti,ab,kf OR (retrospective adj7 (study or studies or design or analysis or analyses or data or review)).ti,ab,kf OR ((case adj control) or (case adj comparison) or (case adj controlled)).ti,ab,kf OR (case-referent adj3 (study or studies or design or analysis or analyses)).ti,ab,kf OR (population adj3 (study or studies or analysis or analyses)).ti,ab,kf OR (descriptive adj3 (study or studies or design or analysis or analyses)).ti,ab,kf OR ((multidimensional or (multi adj dimensional)) adj3 (study or studies or design or analysis or analyses)).ti,ab,kf OR (cross adj sectional adj7 (study or studies or design or research or analysis or analyses or survey or findings)).ti,ab,kf OR ((natural adj experiment) or (natural adj experiments)).ti,ab,kf OR (quasi adj (experiment or experiments or experimental)).ti,ab,kf OR ((non experiment or nonexperiment or non experimental or nonexperimental) adj3 (study or studies or design or analysis or analyses)).ti,ab,kf OR (prevalence adj3 (study or studies or analysis or analyses)).ti,ab,kf OR case series.ti,ab,kf OR case reports.pt OR case reports as topic.sh OR case report.sh OR case study.sh OR (case adj3 (report or reports or study or studies or histories)).ti,ab,kf OR organizational case studies.sh  OR  Exp qualitative research OR focus groups.sh OR interview.sh OR (qualitative* OR focus group* OR interview* OR questionnaire* OR fieldwork OR field stud* OR field research OR narrative* OR discourse analys* OR thematic analys* OR theme* OR content analys* OR grounded theor* OR grounded stud* OR grounded research OR grounded analys* OR lived experience* OR personal experience* OR patient* experience* OR "experience of care" OR care experience* OR perceived quality OR mixed method* OR multiple method* OR ethnograph* OR perspective* OR life story OR life stories OR phenomenol* OR action research* OR case stud* OR conversation analys* OR critical social theor* OR hermeneutic).tw  OR  (systematic review or meta-analysis).pt. OR meta-analysis/ or systematic review/ or systematic reviews as topic/ or meta-analysis as topic/ or "meta analysis (topic)"/ or "systematic review (topic)"/ or exp technology assessment, biomedical/ or network meta-analysis/ OR ((systematic* adj3 (review* or overview*)) or (methodologic* adj3 (review* or overview*))).ti,ab,kf. OR ((quantitative adj3 (review* or overview* or synthes*)) or (research adj3 (integrati* or overview*))).ti,ab,kf. OR ((integrative adj3 (review* or overview*)) or (collaborative adj3 (review* or overview*)) or (pool* adj3 analy*)).ti,ab,kf. OR (data synthes* or data extraction* or data abstraction*).ti,ab,kf. OR (handsearch* or hand search*).ti,ab,kf. OR (mantel haenszel or peto or der simonian or dersimonian or fixed effect* or latin square*).ti,ab,kf. OR (met analy* or metanaly* or technology assessment* or HTA or HTAs or technology overview* or technology appraisal*).ti,ab,kf. OR (meta regression* or metaregression*).ti,ab,kf. OR (meta-analy* or metaanaly* or systematic review* or biomedical technology assessment* or bio-medical technology assessment*).mp,hw. OR (medline or cochrane or pubmed or medlars or embase or cinahl).ti,ab,hw. OR (cochrane or (health adj2 technology assessment) or evidence report).jw. OR (comparative adj3 (efficacy or effectiveness)).ti,ab,kf. OR (outcomes research or relative effectiveness).ti,ab,kf. OR ((indirect or indirect treatment or mixed-treatment or bayesian) adj3 comparison*).ti,ab,kf. OR (multi* adj3 treatment adj3 comparison*).ti,ab,kf. OR (mixed adj3 treatment adj3 (meta-analy* or metaanaly*)).ti,ab,kf. OR umbrella review*.ti,ab,kf. OR (multi* adj2 paramet* adj2 evidence adj2 synthesis).ti,ab,kf. OR (multiparamet* adj2 evidence adj2 synthesis).ti,ab,kf. OR (multi-paramet* adj2 evidence adj2 synthesis).ti,ab,kf.  OR  (Randomized Controlled Trial OR Controlled Clinical Trial OR Pragmatic Clinical Trial OR Clinical Study OR Adaptive Clinical Trial OR Equivalence Trial).pt OR (Clinical Trial OR Clinical Trial, Phase I OR Clinical Trial, Phase II OR Clinical Trial, Phase III OR Clinical Trial, Phase IV OR Clinical Trial Protocol).pt OR Multicenter Study.pt OR Clinical Studies as Topic/ OR exp Clinical Trial/ OR exp Clinical Trials as Topic/ OR Clinical Trial Protocol/ OR Clinical Trial Protocols as Topic/ OR exp "Clinical Trial (topic)"/ OR Multicenter Study/ OR Multicenter Studies as Topic/ OR "Multicenter Study (topic)"/ OR Randomization/ OR Random Allocation/ OR Double-Blind Method/ OR Double Blind Procedure/ OR Double-Blind Studies/ OR Single-Blind Method/ OR Single Blind Procedure/ OR Single-Blind Studies/ OR Placebos/ OR Placebo/ OR Control Groups/ OR Control Group/ OR Cross-Over Studies/ OR Crossover Procedure/ OR (random* OR sham OR placebo*).ti,ab,hw,kf. OR ((singl* OR doubl*) adj (blind* OR dumm* OR mask*)).ti,ab,hw,kf. OR ((tripl* OR trebl*) adj (blind* OR dumm* OR mask*)).ti,ab,hw,kf. OR (control* adj3 (study OR studies OR trial* OR group*)).ti,ab,hw,kf. OR (clinical adj3 (study OR studies OR trial*)).ti,ab,hw,kf. OR (Nonrandom* OR non random* OR non-random* OR quasi-random* OR quasirandom*).ti,ab,hw,kf. OR (phase adj6 (study OR studies OR trial*)).ti,ab,hw,kf. OR ((crossover OR cross-over) adj3 (study OR studies OR trial*)).ti,ab,hw,kf. OR ((multicent* OR multi-cent*) adj3 (study OR studies OR trial*)).ti,ab,hw,kf. OR allocated.ti,ab,hw. OR ((open label OR open-label) adj5 (study OR studies OR trial*)).ti,ab,hw,kf. OR ((equivalence OR superiority OR non-inferiority OR noninferiority) adj3 (study OR studies OR trial*)).ti,ab,hw,kf. OR (pragmatic study OR pragmatic studies).ti,ab,hw,kf. OR ((pragmatic OR practical) adj3 trial*).ti,ab,hw,kf. OR ((quasiexperimental OR quasi-experimental) adj3 (study OR studies OR trial*)).ti,ab,hw,kf. OR trial.ti,kf OR ((prepost OR pre-post) adj3 stud*).ti,ab,kf OR (interrupt* adj3 time adj3 series).ti,ab,kf OR (before adj3 after adj3 series).ti,ab,kf | 11684640 |
| 4 | # 1 AND #2 AND #3 | 7278 |
| 5  Animal Studies | ((exp animals) NOT (exp animals AND exp humans)) | 5308398 |
| 6 | #4 NOT #5 | 7111 |

# EGM Framework

- POPULATION [Not selectable (no checkbox)]
  - People who are living with MND [Selectable (show checkbox)]
    - MND/Amyotrophic lateral sclerosis [Selectable (show checkbox)]
    - Progressive Bulpar Palsy [Selectable (show checkbox)]
    - Progressive muscular atrophy [Selectable (show checkbox)]
    - Primary lateral sclerosis [Selectable (show checkbox)]
    - MND with frontal temporal dementia [Selectable (show checkbox)]
    - Kennedy’s disease- spinal and bulbar muscular atrophy (SBMA). [Selectable (show checkbox)]
  - Asymptomatic Genetic Carriers [Selectable (show checkbox)]
    - C9orf72 [Selectable (show checkbox)]
    - SOD1 [Selectable (show checkbox)]
    - FUS [Selectable (show checkbox)]
    - TARDBP [Selectable (show checkbox)]
    - Not specified [Selectable (show checkbox)]
    - Genetically at risk [Selectable (show checkbox)]
  - Carers [Selectable (show checkbox)]
  - Family members and friends of people who are living with MND [Selectable (show checkbox)]
  - Healthcare professionals [Selectable (show checkbox)]
    - Health care professionals [Selectable (show checkbox)]
    - Support workers [Selectable (show checkbox)]
  - Communities of Focus [Selectable (show checkbox)]
    - First Nations Peoples [Selectable (show checkbox)]
    - Culturally and Linguistically Diverse Peoples [Selectable (show checkbox)]
    - Remote/Rural/Regional Specific [Selectable (show checkbox)]
    - Australian-specific trial [Selectable (show checkbox)]
- TYPE OF STUDY [Not selectable (no checkbox)]
  - RCTS [Selectable (show checkbox)]
  - Non-randomised studies of interventions [Selectable (show checkbox)]
  - Systematic reviews and overviews [Selectable (show checkbox)]
  - Qualitative studies [Selectable (show checkbox)]
  - Trial- CONFIRMED ONGOING [Selectable (show checkbox)]
- INTERVENTIONS [Not selectable (no checkbox)]
  - Asymptomatic Genetic Carrier-Specific [Intervention]
    - Genetic Counselling [Intervention]
    - presymptomatic monitoring [Intervention]
    - Early Referral [Intervention]
    - Preventative treatments [Intervention]
  - Carer-specific [Intervention]
    - Respite Care [Selectable (show checkbox)]
    - Peer-support [Selectable (show checkbox)]
    - Counselling [Selectable (show checkbox)]
    - Palliative Rehabilitation [Selectable (show checkbox)]
  - Complementary Therapies [Intervention]
    - Acupuncture [Intervention]
    - Bee Venom [Intervention]
    - Bowen Technique [Intervention]
    - Neurolinguistic Programming [Intervention]
  - Education & Awareness [Intervention]
    - Self-Care for Carers [Intervention]
    - Skills Training & Support Programs [Intervention]
    - Impact of Survival Prediction Models [Intervention]
  - End-of-Life Care [Intervention]
    - Advanced Care Planning [Intervention]
    - Assisted Dying [Intervention]
    - Bereavement Care [Intervention]
    - Naming of Health-Care Proxy [Intervention]
  - Medical Devices, adaptive equipment & Assistive Technologies [Intervention]
    - Communication Devices [Intervention]
      - Alphabet [Intervention]
      - call bell or personal alarms [Intervention]
      - C/tablet-based voice output [Intervention]
      - computers [Intervention]
      - computerized speech synthesizers [Intervention]
      - eye-gaze devices [Intervention]
      - hands-free telephone [Intervention]
      - laser head pointer [Intervention]
      - neurolink brain chip [Intervention]
      - Perspex eye-gaze frame (ETRAN board) [Intervention]
      - picture boards [Intervention]
      - scanners [Intervention]
      - smart phones [Intervention]
      - Switches [Intervention]
      - tablets [Intervention]
      - telephone typewriter (TTY) [Intervention]
      - text to speech apps [Intervention]
      - voice activated devices [Intervention]
      - voice amplifications [Intervention]
      - voice and message banking [Intervention]
      - Brain-computer interface [Selectable (show checkbox)]
      - text to speech devices [Intervention]
      - general communication device use [Intervention]
    - Home equipment [Intervention]
      - Commodes [Intervention]
      - environmental control systems [Intervention]
      - grab rails [Intervention]
      - hospital (electric) beds [Intervention]
      - hoists [Intervention]
      - pressure care mattresses [Intervention]
      - riser–recliner chairs [Intervention]
      - small ramps [Intervention]
    - Home modifications [Intervention]
      - stairlifts [Intervention]
      - lifts [Intervention]
      - shower fittings [Intervention]
      - home renovations [Intervention]
    - Mobility Aids [Intervention]
      - Armon edero-mechanical arm [Intervention]
      - exoskeleton [Intervention]
      - manual wheelchairs [Intervention]
      - mobility aids [Intervention]
      - power wheelchairs [Intervention]
      - tilt-in-space wheelchair [Intervention]
      - walking sticks [Intervention]
      - frames [Intervention]
    - Orthoses [Intervention]
      - Braces [Intervention]
      - Halo HCTO [Intervention]
      - head support [Intervention]
      - headmaster collar [Intervention]
      - Minerva CTO [Intervention]
      - orthotics [Intervention]
      - soft and hard cervical collars [Intervention]
      - splint [Intervention]
      - upper extremity orthotics [Intervention]
      - write hand orthosis. [Intervention]
    - Wearable Technology [Intervention]
      - Accelerometers [Intervention]
  - Management of Care & Service Models [Intervention]
    - Aged Care services [Intervention]
    - Community-based Care [Intervention]
    - Models of Care [Intervention]
      - Continuity of care [Intervention]
      - Multidisciplinary clinic care [Intervention]
      - Network-based model [Intervention]
      - MND specific clinic [Intervention]
    - Palliative Care [Intervention]
    - Short term accomodation [Intervention]
    - Telemonitoring [Intervention]
      - Mobile Health (mHealth) [Intervention]
      - Remote Patient Monitoring (RPM) [Intervention]
      - Careportal [Intervention]
    - Telehealth [Intervention]
    - Tertiary (Hospital) [Intervention]
    - Expanded Access Protocol (EAP) [Intervention]
  - Pharmaceutical [Intervention]
    - Antidepressants [Intervention]
      - Tricyclic antidepressants (amitriptyline, mitriptyline, trazodone) [Intervention]
      - citalopram [Intervention]
      - Ezogabine (also known as Retigabine [Intervention]
    - antiemetic [Intervention]
      - Anticholinergic agents (Hyoscine, Glycopyrronium Bromide) [Intervention]
      - tetracannabinoid (THC) [Intervention]
    - Antitussive (Cough suppressants) [Intervention]
      - Opiods [Codeine] [Intervention]
      - Nonopiods [Dextromethorphan] [Intervention]
      - Mucolytics (N-acetylcysteine, Carbocistene) [Selectable (show checkbox)]
    - Immunomodulatory Therapies [Intervention]
      - Immunosupressants (Azathiprine, Cyclosporine, abatacept (CTLA4-Ig)) [Intervention]
      - butyrates [Intervention]
      - corticosteroids [Intervention]
      - H.P. Acthar® Gel [Intervention]
      - Lymphoid Irradiation [Intervention]
      - NP001 (sodium chlorite) [Intervention]
      - Monoclonal antibodies (rituximab,ozanezumab) [Intervention]
      - WF10 (Sodium Chlorite) [Intervention]
      - levamisole (antihelminthic) [Selectable (show checkbox)]
      - Interleukin 2 [Selectable (show checkbox)]
      - Ursodeoxycholic Acid [Intervention]
      - aldesleukin [Intervention]
    - mood stabilizer [Intervention]
      - Cannabis [Psychodelic] [Intervention]
      - CBD oil [Intervention]
      - levetiracetam [primarily anticonvulsants] [Intervention]
      - Lithium [Intervention]
      - Modafinil [Psychostimulant] [Intervention]
      - Psilocybin [Psychodelics] [Intervention]
    - muscle relaxants [Intervention]
      - Baclofen [GABA mimetic] [Intervention]
      - botulinum neurotoxin [Intervention]
      - Chlorzoxazone [Mephenesin analouge] [Intervention]
      - Dantrolene sodium [Intervention]
      - Benzodiazepines (Diazepam, Tetrazepam) [Intervention]
      - Tizanidine [α2 agonist] [Intervention]
      - Zolpidem [selective benzodiazepine receptor agonist] [Intervention]
      - Quinidine [Intervention]
      - Mexiletine [Outcome]
      - TJ-68 (Shakuyakukanzoto) [Selectable (show checkbox)]
      - tirasemtiv [Intervention]
      - antimuscarinics (Scopolamine) [Intervention]
      - CK-2017357 [Intervention]
    - Neuroprotective [Intervention]
      - Arimoclomol [Intervention]
      - Brain-derived neurotrophic factor (BDNF) [Intervention]
      - Ciliary neurotrophic factor (CNTF) [Intervention]
      - Selective estrogen receptor modulator (Clomiphene, Tamoxifen, Raloxifen) [Intervention]
      - CNM-Au8 [Intervention]
      - GM604 [Intervention]
      - Glatiramer acetate [Intervention]
      - IFB-088 Plus [Intervention]
      - Insulin like growth factor 1 (IGF-1) [Intervention]
      - Glutamate-blocking agent (Riluzole, Gabapentin, Perampanel) [Intervention]
      - RT001 [Intervention]
      - Toforsen [Intervention]
      - Xaliproden [Intervention]
      - Valproate (Valproic acid; anticonvulsant) [Selectable (show checkbox)]
      - Dexpramipexole [Selectable (show checkbox)]
      - Masitinib (AB10015; anticancer drug) [Selectable (show checkbox)]
      - Free radical scavenger (Edaravone, Vitamin C) [Selectable (show checkbox)]
      - Monepantel (anthelminthic) [Selectable (show checkbox)]
      - Myeloperoxidase Inhibitors (Verdiperstat) [Selectable (show checkbox)]
      - Omigapil (TCH346) [Intervention]
      - Mecasin [Intervention]
      - Recombinant Human HGF (KP-100) [Intervention]
      - sodium phenylbutyrate-taurursodiol [Intervention]
      - Memantine [Intervention]
      - G-CSF (Granulocyte Colony-Stimulating Factor) [Intervention]
      - Antioxidant (e.g Edaravone) [Intervention]
      - 3K3A-APC [Intervention]
      - Tetramethylpyrazine Nitrone [Intervention]
      - Flecainide [Intervention]
      - Rasagiline (monoamine oxidase inhibitor) [Intervention]
      - PrimeC (celecoxib and ciprofloxacin) [Intervention]
      - Anticoagulant (e.g ILB) [Intervention]
      - Statin [Intervention]
      - Nanocurcumin [Intervention]
      - Bile Acid Salts (Tauroursodeoxycholic acid) [Intervention]
      - GEMALS (Endotherapia) [Intervention]
      - antisense oligonucleotide BIIB078 [Intervention]
      - mEphA4-Fc [Intervention]
      - Ibudilast (MN-166) [Intervention]
    - pseudobulbar affect [Intervention]
      - NUEDEXTA [Intervention]
    - sedative [Intervention]
      - Benzodiazepines (Diazepam, tetrazepam) [Intervention]
      - Propofol [Anaesthetic] [Intervention]
    - Administrative methods of phamaceutical [Intervention]
      - intravenous (IV) [Intervention]
      - intramuscular [Intervention]
      - subcatenously [Intervention]
      - Syringe drivers [Intervention]
      - Intrathecal [Selectable (show checkbox)]
      - Oral [Selectable (show checkbox)]
    - Bronchodilator [Outcome]
      - Beta2 agonist (Salbutamol, Clenbuterol) [Outcome]
    - Vasodilator [Selectable (show checkbox)]
      - phosphodiesterase inhibitor (pentoxifylline) [Selectable (show checkbox)]
      - guanabenz acetate [Intervention]
    - Hormone derivative/analogue [Intervention]
      - thyrotropin-releasing hormone (TRH) [Intervention]
      - Erythropoietin (EPO) [Intervention]
      - luteinising hormone-releasing hormone agonists (leuprorelin) [Intervention]
    - Antihypertensive [Selectable (show checkbox)]
      - Calcium channel blocker (nimodipine, verapamil) [Selectable (show checkbox)]
    - Skeletal muscle activator (reldesemtiv, tirasemtiv, levosimendan) [Intervention]
    - Antibiotics/antivirals [Selectable (show checkbox)]
      - Cephalosporin (Ceftriaxone) [Selectable (show checkbox)]
      - Antiretroviral [Selectable (show checkbox)]
      - Fluroquinolone (Ciprofloxacin) [Selectable (show checkbox)]
      - Penicillin G/Hydrocortisone [Intervention]
    - Anti-inflammatory (celecoxib) [Selectable (show checkbox)]
    - Vitamins/minerals [Intervention]
  - Nutrition & Dietary Support [Intervention]
    - Dietary Interventions [Intervention]
      - Deanna Protocol [Intervention]
      - fluid intake [Intervention]
      - Gluten-free diet [Intervention]
      - High carbohydrate hypercaloric diet [Intervention]
      - High-fat hypercaloric diet [Intervention]
      - High protein / high energy diet (ie. HPHE) [Intervention]
      - Isocaloric tube-fed diet [Intervention]
      - Ketogenic diet (including Coconut Oil [Intervention]
      - Medium chain triglycerides [Intervention]
      - Oral Nutritional supplements [Intervention]
      - WAHLS protocol. [Intervention]
      - creatine supplementation [Selectable (show checkbox)]
      - Amino Acid Supplementation [Selectable (show checkbox)]
    - Invasive nutritional interventions [Intervention]
      - Home (or total) Parenteral Nutrition (HPN) [Intervention]
      - Nasogastric Tube (NGT) [Intervention]
      - Per-Oral Image-Guided Gastrostomy (PIG) [Intervention]
      - Percutaneous Endoscopic Gastrostomy (PEG) [Intervention]
      - Radiologically Inserted Gastrostomy (RIG) [Intervention]
      - Gastrostomy [Selectable (show checkbox)]
      - Computated Tomography-Guided Percutaneous Gastrostomy (CPG) [Intervention]
    - Swallowing management [Intervention]
      - direct / indirect swallowing techniques (eg. chin tilt [Intervention]
      - supraglottic swallow) [Intervention]
      - food/fluid texture modification [Intervention]
    - Non- Invasive Nutritional Support [Selectable (show checkbox)]
      - Pharyngeal electrical stimulation [Selectable (show checkbox)]
    - Fat supplementation (eg Lecithin) [Intervention]
    - Nutritional Counselling [Intervention]
      - Nurse Dietary Counselling [Intervention]
      - Dietician [Intervention]
  - Psychological and behavioural therapy [Intervention]
    - Education, Counseling and Social Support [Intervention]
      - group therapy [Intervention]
      - peer connection/support [Intervention]
      - online forum [Intervention]
      - Psychoeducational [Intervention]
      - Energy conservation [Selectable (show checkbox)]
    - Expressive & Creative Therapies [Intervention]
      - Animal therapy [Intervention]
      - art therapy [Intervention]
      - dance therapy [Intervention]
      - drama therapy [Intervention]
      - expressive disclosure [Intervention]
      - music therapy [Intervention]
      - writing therapy. [Intervention]
    - Family Support [Intervention]
      - Family Therapy [Intervention]
    - Mindfulness & Relaxation Techniques [Intervention]
      - hypnosis [Intervention]
      - meditation [Selectable (show checkbox)]
      - mindfulness [Selectable (show checkbox)]
    - Psychotherapeutic Approaches [Intervention]
      - Acceptance and Commitment Therapy (ACT) [Intervention]
      - Cognitive Behavioral Therapy (CBT) [Intervention]
      - Counselling [Intervention]
      - Dignity Therapy (DT) [Intervention]
      - Empathy-based supportive counseling [Intervention]
      - Eye movement desensitization and reprocessing (EMDR) [Intervention]
      - Psycho-therapeutic Nursing [Intervention]
      - Psychotherapy. [Intervention]
      - self-compassion interventions (Compassionate Mind Training) [Intervention]
    - Specialized & Supportive Therapies [Intervention]
      - Genetic counselling [Selectable (show checkbox)]
      - speech therapy [Intervention]
  - Physical & Rehabilitation Interventions [Intervention]
    - Circulatory Support [Intervention]
      - compression stockings [Intervention]
      - pneumatic compression devices (PCDs) [Intervention]
      - repositioning [Intervention]
    - Pressure Injury Prevention and Management [Intervention]
      - Repositioning [Intervention]
      - moisturising [Intervention]
      - wound care [Intervention]
      - dressings [Intervention]
      - debridement [Intervention]
      - wound cleansing and irrigation [Intervention]
      - negative pressure wound therapy (NPWT) [Intervention]
      - hyperbaric oxygen therapy (HBOT) [Intervention]
      - compression stockings [Intervention]
      - pneumatic compression devices (PCDs) [Intervention]
    - Physical Therapy & Rehabilitation [Intervention]
      - Biofeedback [Intervention]
      - casting [Intervention]
      - chemodenervation [Intervention]
      - electrical stimulation [Intervention]
      - endurance exercise [Intervention]
      - falls prevention [Intervention]
      - fatigue management [Intervention]
      - hydrotherapy [Intervention]
      - icebaths [Intervention]
      - lymphatic massage [Intervention]
      - massage (remedial) [Intervention]
      - massage (therapeutic) [Intervention]
      - neurophysio [Intervention]
      - podiatry care [Intervention]
      - pressure garments [Intervention]
      - recovery boots [Intervention]
      - sauna [Intervention]
      - stretching [Intervention]
      - submaximal exercise [Intervention]
      - ultrasound [Intervention]
      - positioning [Intervention]
      - physical exercise [Intervention]
      - heat/cold therapy [Intervention]
      - MotorMed. [Intervention]
      - Aerobic exercise [Selectable (show checkbox)]
      - Resistance training [Selectable (show checkbox)]
      - TRAIN program [Selectable (show checkbox)]
      - Robot-assisted gait training (RAGT) [Intervention]
      - Osteopathic Manual Treatment (OMT) [Intervention]
      - Physiotherapy [Intervention]
      - Occupational Therapy [Intervention]
  - Respiratory management [Intervention]
    - Cough assist [Intervention]
      - cough augmentation [Intervention]
      - cough assist devices [Intervention]
      - mechanical in-exsufflator (MI-E) for cough assistance [Intervention]
      - Tussive squeeze [Intervention]
      - Manual Cough Assist Technique [Intervention]
    - General respiratory support [Intervention]
      - Humidification [Intervention]
      - nebulisers [Intervention]
      - carbocisteine [Intervention]
      - electric fans [Intervention]
      - respiratory strength training [Intervention]
      - electrical stimulation of the diaphragm [Intervention]
      - unassisted breath stacking [Intervention]
      - assisted breath stacking (for example [Intervention]
      - using a lung volume recruitment bag [Intervention]
      - air stacking) [Intervention]
      - suction [Intervention]
      - mechanical insufflator-exsufflators [Intervention]
      - sleep positioning [Intervention]
      - oxygen converter. [Intervention]
      - Lung Volume Recruitment Training [Intervention]
      - Yoga-style Diaphragmatic Breathing [Intervention]
    - Non-invasive ventilatory support [Intervention]
      - Bilevel positive airway pressure [Intervention]
      - Continuous positive airway pressure [Intervention]
      - mask wearing [Intervention]
      - ‘sip-and-puff’ ventilation [Intervention]
      - withdrawing NIV [Intervention]
      - oxygen therapy [Intervention]
      - noninvasive positive pressure ventilation (NIPPV). [Intervention]
      - NIV [Intervention]
      - Volume-assured pressure support (VAPS) [Intervention]
      - Pressure Support (PS) [Intervention]
    - Invasive Ventilatory support [Intervention]
      - Tracheostomy [Intervention]
      - mechanical ventilation [Intervention]
      - translaryngeal intubation [Intervention]
      - laryngectomy [Intervention]
      - NeuRx® Diaphragm Pacing System™ (DPS) [Intervention]
      - laryngeal closure [Intervention]
      - withdrawing IV [Intervention]
  - Social Services/ Financial [Intervention]
    - Financial support and advice [Intervention]
    - Social services [Intervention]
      - Aged care support [Intervention]
      - money management [Intervention]
      - benefits and grants [Intervention]
      - continuing healthcare funding [Intervention]
      - funeral expenses. [Intervention]
    - NDIS Support [Intervention]
  - Surgical interventions [Intervention]
    - anesthesia [Intervention]
    - General surgery [Selectable (show checkbox)]
    - Cerebrospinal fluid filtration [Intervention]
    - Diaphragm pacing [Intervention]
  - Non-invasive brain stimulation [Selectable (show checkbox)]
  - Transfusion/transplantation [Selectable (show checkbox)]
    - Stem cell/mononuclear cells transplantation [Selectable (show checkbox)]
    - Plasma exchange/Plasmapheresis [Selectable (show checkbox)]
    - Fetal olfactory ensheathing cells [Selectable (show checkbox)]
    - Regulatory T-Lymphocyte (Treg) [Intervention]
    - Fecal Microbiota [Intervention]
    - Allogeneic Adipose Tissue Mesenchymal Stromal Cells [Intervention]
  - Radiotherapy [Intervention]
- OUTCOMES [Not selectable (no checkbox)]
  - Adherence to Interventions & Treatment Satisfaction [Selectable (show checkbox)]
    - Quality of care [Selectable (show checkbox)]
    - Patient Engagement [Selectable (show checkbox)]
    - Adherence [Selectable (show checkbox)]
  - awareness & knowledge of MND care [Selectable (show checkbox)]
    - Patient & Caregiver Education [Selectable (show checkbox)]
    - Healthcare provider knowledge [Selectable (show checkbox)]
  - cognitive function & neurological decline [Selectable (show checkbox)]
    - Cognitive Symptoms [Selectable (show checkbox)]
    - Emotional Lability [Selectable (show checkbox)]
  - economic cost and health service outcomes. [Selectable (show checkbox)]
    - Financial Impact [Selectable (show checkbox)]
    - Implementation Cost (Proctor) [Selectable (show checkbox)]
    - Health service utilisation outcome [Selectable (show checkbox)]
    - Initiation to intervention [Selectable (show checkbox)]
      - Initiation to tracheostomy [Selectable (show checkbox)]
      - Initiation to gastrostomy [Selectable (show checkbox)]
  - Implementation & Contextual Factors (Proctor’s Outcomes Integrated) [Selectable (show checkbox)]
    - Acceptability [Selectable (show checkbox)]
    - adoption [Selectable (show checkbox)]
    - appropriateness [Selectable (show checkbox)]
    - equity-focused care [Selectable (show checkbox)]
    - feasibility [Selectable (show checkbox)]
    - fidelity [Selectable (show checkbox)]
    - patient & caregiver experiences [Selectable (show checkbox)]
    - penetration [Selectable (show checkbox)]
    - sustainability. [Selectable (show checkbox)]
  - psychological & emotional wellbeing [Selectable (show checkbox)]
    - Cognitive & Behavioral Aspects [Selectable (show checkbox)]
    - Emotional & Social Factors [Selectable (show checkbox)]
    - Mental Health & Coping [Selectable (show checkbox)]
  - Physical health & disease progression [Selectable (show checkbox)]
    - Constipation [Selectable (show checkbox)]
    - cramping and muscle spasm [Selectable (show checkbox)]
    - gastrointestinal issues [Selectable (show checkbox)]
    - general progression [Selectable (show checkbox)]
    - infections & complications [Selectable (show checkbox)]
    - neuromuscular symptoms [Selectable (show checkbox)]
    - ALS Functional Rating Scale (ALSFRS-R) [Selectable (show checkbox)]
    - Time to NIV [Selectable (show checkbox)]
    - Time to gastrostomy [Selectable (show checkbox)]
    - Biological indicators/biomarkers [Selectable (show checkbox)]
    - daily functioning and overall wellbeing [Selectable (show checkbox)]
    - Spasticity [Selectable (show checkbox)]
  - quality of life & symptoms [Selectable (show checkbox)]
    - Communication [Selectable (show checkbox)]
    - Sleep [Selectable (show checkbox)]
    - nutrition [Selectable (show checkbox)]
    - pain [Selectable (show checkbox)]
    - psychosocial impact [Selectable (show checkbox)]
    - saliva control (sialorrhoea) [Selectable (show checkbox)]
    - symptom management. [Selectable (show checkbox)]
    - Quality of Life [Selectable (show checkbox)]
    - Fatigue [Selectable (show checkbox)]
  - respiratory function & related complications [Selectable (show checkbox)]
    - dyspnea [Selectable (show checkbox)]
    - respiratory function. [Selectable (show checkbox)]
    - Ventilatory support [Selectable (show checkbox)]
    - Tracheostomy [Selectable (show checkbox)]
  - spiritual wellbeing & dignity [Selectable (show checkbox)]
    - Spiritual Aspects [Selectable (show checkbox)]
  - Survival & Mortality Outcomes [Selectable (show checkbox)]
    - Life Expectancy Measures [Selectable (show checkbox)]
    - Time to tracheostomy [Selectable (show checkbox)]
  - Adverse Events [Selectable (show checkbox)]
  - Weight Outcomes [Selectable (show checkbox)]
    - Body Mass Index (BMI) [Selectable (show checkbox)]
    - Body Fat-Free Mass [Selectable (show checkbox)]
